# Prior exercise does not affect chylomicron particle number following a mixed meal of moderate fat content

**DOI:** 10.1186/1476-511X-6-8

**Published:** 2007-03-30

**Authors:** Anthony P James, Karin Slivkoff-Clark, John CL Mamo

**Affiliations:** 1 School of Public Health, Australian Technology Centre for Metabolic Fitness, Curtin University of Technology, Perth, WA, Australia

## Abstract

**Background:**

A single session of exercise has been reported to reduce fasting and postprandial triacylglycerol concentrations on the subsequent day. It is possible that exercise also reduces chylomicron particle number, which may underlie the observed reduction in postprandial triacylglycerol concentration. In the present study we aimed to determine whether a single session of exercise reduces fasting and postprandial chylomicron particle number on the subsequent day. In a randomised crossover design eight lean and healthy male and female subjects attended two postprandial testing days. On the previous day the subjects either performed 90 minutes of moderate intensity exercise or did not perform any exercise. Fasting blood samples were then collected prior to ingestion of a moderate fat mixed meal (0.44 g fat, 0.94 g carbohydrate, 0.27 g protein/kg body weight), blood was then collected after 1 h, 2 h, 4 h, 6 h, and 8 h.

**Results:**

The fasting and postprandial apolipoprotein B48 concentration (marker of chylomicron particle number) was not affected by prior exercise. However exercise reduced fasting triacylglycerol concentration by 16% (*P *< 0.05) and there was a trend towards a reduction in the total area under the postprandial triacylglycerol curve (23%; *P *= 0.053). However when corrected for baseline concentration postprandial triacylglycerol concentration was not affected by prior exercise.

**Conclusion:**

A single session of exercise of moderate intensity and 90 minutes duration reduces fasting triacylglycerol levels, however fasting and postprandial chylomicron particle number was unaffected. Furthermore it appears that previously observed reductions in postprandial triacylglycerol levels following exercise are only mediated following consumption of high, non-physiologically relevant doses of fat.

## Background

Many studies have reported beneficial effects of exercise in reducing risk factors for a range of diseases including cardiovascular disease (CVD). Until recently the majority of studies have examined the effects of a chronic exercise intervention on fasting CVD risk factors. Although the results from such studies have been variable the most consistent findings are that an exercise intervention leads to an increase in HDL cholesterol and a reduction in fasting triacylglycerol [1-3]. Numerous studies have observed that performing a single session of exercise leads to a reduction in both fasting and postprandial triacylglycerol concentration following consumption of a high fat load on the day subsequent to the exercise session [4,5].

We have reported that obese, insulin resistant individuals exhibit elevated fasting and postprandial chylomicron particle number [6] and there is increasing evidence that chylomicron remnants are able to penetrate, and hence contribute cholesterol to the arterial wall [7]. It is possible that the mechanisms responsible for the reduction in fasting and postprandial triacylglycerol concentrations following exercise also lead to a reduction in chylomicron concentration. The exercise-induced reduction in postprandial lipaemia may be due, at least in part, to an increased lipolysis or clearance of chylomicron-associated triacylglycerol. As a consequence of this improved chylomicron metabolism we may also predict a decrease in chylomicron particle number following exercise. In support of this the total lipoprotein concentration in a chylomicron rich lipoprotein fraction has been reported to be reduced following exercise [8]. However the interpretation of such measurements may be confounded by the presence of hepatically derived lipoproteins in the chylomicron rich fraction and/or the existence of chylomicron particles in other, more dense, lipoprotein fractions. By specifically measuring apolipoprotein (apo) B48 concentration in serum we are able to determine the specific effect of exercise on chylomicron particle number.

We have aimed to investigate whether a single session of exercise of the duration and intensity previously reported to reduce fasting and postprandial triacylglycerol concentrations also leads to a reduction in chylomicron particle number and hence may further reduce CVD risk. In order to allow comparison to the previous studies we have also used young healthy subjects who are not overweight or obese.

## Results

The subject characteristics are indicated in Table 1. Subjects were young (29.8 ± 2.0 years), not overweight or obese (BMI = 20.8 ± 1.0 kg/m^2^).

**Table 1:** Baseline subject characteristics

***Characteristic***	***Mean***
Age (y)	29.8 (2.0)
Weight (kg)	61.0 (4.9)
Height (cm)	170.4 (3.3)
BMI (kg/m^2^)	20.8 (1.0)
Waist circumference (cm)	72.4 (3.6)

Shown are means and standard errors (in brackets) for each parameter. Abbreviations: BMI, Body mass index.

### Dietary Standardisation

The dietary standardisation for three days prior to each postprandial test day was well adhered to. Subjects replicated their diet for the subsequent dietary standardisation with no difference in macronutrient composition and only minor variations in the reported time of consumption of various meals (Table 2).

**Table 2:** Energy and macronutrient content of standard diets

	Control	Exercise
Energy Intake (kJ)	7872 (517)	7931 (527)
Carbohydrate Intake (g)	266 (13)	271 (14)
Protein Intake (g)	87.5 (5.1)	87.6 (5.1)
Total Fat Intake (g)	52.5 (6.1)	53.2 (6.2)
Saturated Fat Intake (g)	23.3 (2.3)	23.6 (2.3)

Shown are means and standard errors (in brackets) of the daily energy and macronutrient content of the standard diets consumed for the control and exercise trials. No significant differences were found between the diets.

### Exercise Session

During the exercise session subjects exercised at an average RPE level of 13.3 ± 0.3 which was within the requested level of 12–14 and corresponds to the descriptor "somewhat hard". The average energy expenditure during the total 90 minutes of exercise was 2426 ± 230 kJ. Average heart rate during the exercise session was 123.3 ± 4.7 beats/min which corresponds to an average HR_max _of 65 ± 2.2%. Blood lactate concentration during the exercise session was on average 4.2 ± 0.6 mM.

### Fasting and Postprandial Lipid Measures

**Figure 1:**
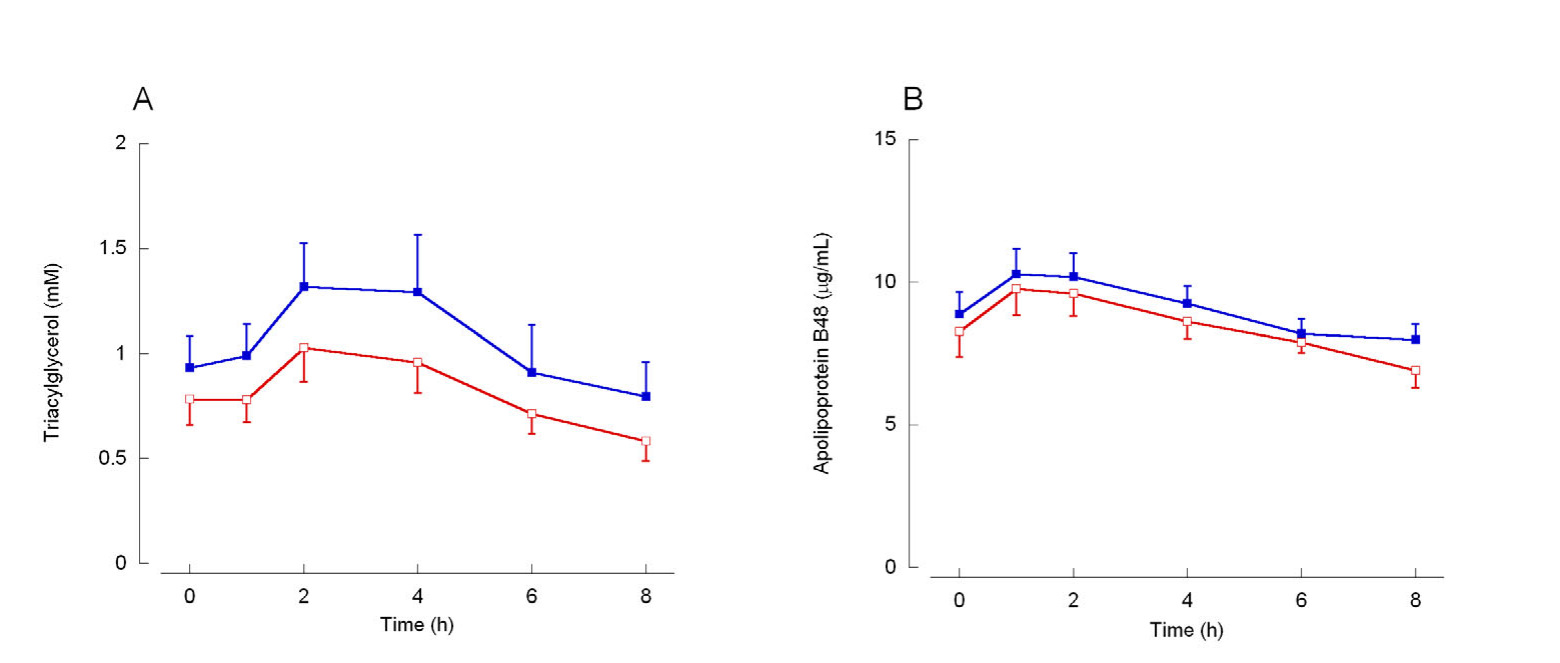
**Serum concentrations of triacylglycerol and apolipoprotein B48 in the fasting and postprandial states**. Shown are the concentrations, mean and standard error of triacylglycerol (A) and apolipoprotein B48 (B) in the fasting state (0 h) and for 8 h after consumption of the moderate fat mixed meal in control (closed squares; blue) and prior exercise (open squares; red) trials. Summary measures are shown in Table 3.

The fasting serum apo B48 concentration was not affected by prior exercise (Figure 1, Table 3). Following consumption of the test meal no change in either the total or incremental areas under the postprandial apo B48 curves was observed (Figure 1, Table 3).

**Table 3:** Fasting and postprandial measures on day subsequent to control or exercise trials

	Control	Exercise
**Fasting**		
Triacylglycerol (mM)	0.93 (0.15)	0.78 (0.12)*
Total Cholesterol (mM)	4.3 (0.2)	4.2 (0.2)
LDL Cholesterol (mM)	2.3 (0.2)	2.5 (0.3)
HDL Cholesterol (mM)	1.6 (0.1)	1.5 (0.1)
Glucose (mM)	5.1 (0.4)	5.2 (0.2)
Insulin (mIU/L)	6.8 (0.3)	6.0 (0.5)
HOMA Score	1.5 (0.1)	1.4 (0.1)
NEFA (mM)	0.37 (0.03)	0.39 (0.03)
Apolipoprotein B48 (μg/mL)	8.9 (0.8)	8.3 (0.9)
**Postprandial**		
Triacylglycerol AUC (mM.h)	8.6 (1.6)	6.6 (0.9)
Triacylglycerol IAUC (mM.h)	1.9 (0.8)	1.2 (0.2)
Apolipoprotein B48 AUC (μg/mL.h)	72.9 (5.1)	68.3 (4.6)
Apolipoprotein B48 IAUC (μg/mL.h)	5.8 (0.6)	8.6 (2.5)
Insulin AUC (mIU/L.h)	75.7 (8.0)	70.1 (9.8)
Insulin IAUC (mIU/L.h)	29.8 (7.2)	28.7 (7.6)
NEFA AUC (mM.h)	3.5 (0.4)	3.5 (0.2)

Shown are means and standard errors (in brackets) for each parameter. Exercise values that were significant different to control values are indicated by * (P < 0.05). Abbreviations: LDL, Low-density lipoprotein; HDL, High-density lipoprotein; HOMA, Homeostatic Model Assessment; AUC, Area under the curve; IAUC, Incremental area under the curve (corrected for fasting concentration).

The fasting triacylglycerol concentration was reduced by 16% following the exercise session (*P *< 0.05; Figure 1, Table 3). Following consumption of the test meal there was a trend towards a reduction in the total area under the postprandial triacylglycerol curves following exercise (23%, *P *= 0.053, Figure 1, Table 3). The incremental area under the triacylglycerol curve (area under the curve corrected for baseline concentration) was not affected by prior exercise.

Following the exercise session there was no difference in fasting total-, LDL- or HDL-cholesterol concentrations compared to control measurements (Table 3). Postprandial cholesterol concentrations were also not affected by prior exercise (data not shown).

**Figure 2:**
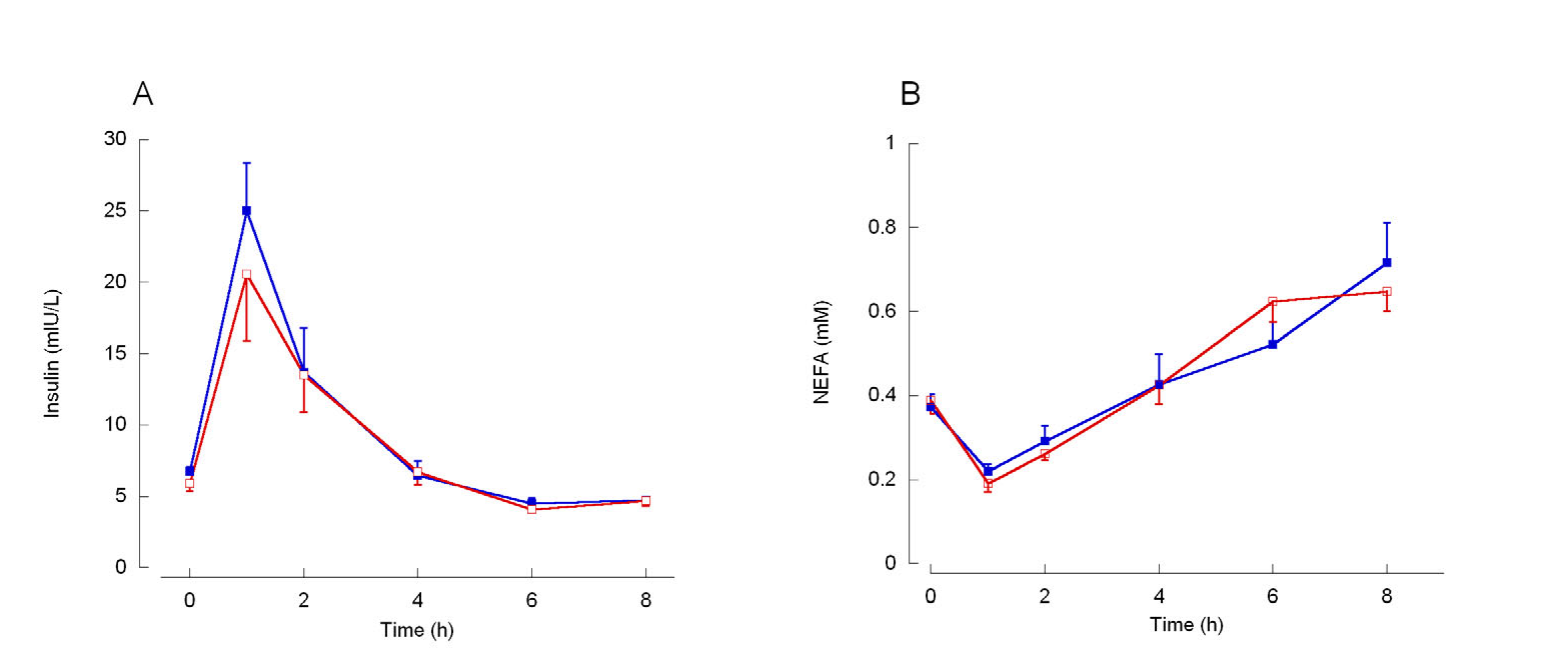
**Serum concentrations of insulin and non-esterified fatty acids (NEFA) in the fasting and postprandial states**. Shown are the concentrations, mean and standard error of insulin (A) and NEFA (B) in the fasting state (0 h) and for 8 h after consumption of the moderate fat mixed meal in control (closed squares; blue) and prior exercise (open squares; red) trials. Summary measures are shown in Table 3.

Measures of insulin sensitivity were not affected by prior exercise with no change in fasting insulin and glucose concentrations or HOMA score after exercise (Table 3). Following the mixed meal insulin concentration increased rapidly before returning to baseline at 4 h. However both the total and incremental areas under the postprandial insulin curves were not affected by prior exercise (Figure 2, Table 3).

The fasting serum NEFA concentration was not different following exercise compared to control. Following consumption of the mixed moderate fat meal, serum NEFA concentrations were initially suppressed at 1–2 h before increasing and remaining elevated at 8 h postprandial (Figure 2). The total area under the postprandial NEFA curve was not affected by prior exercise (Table 3). Incremental area under the curve was not calculated as a measure of postprandial NEFA as this curve displayed a suppression below baseline (Figure 2).

## Discussion

In agreement with a range of previous studies we report that a single session of moderate intensity exercise decreases fasting triacylglycerol concentration on the subsequent day. There was a trend towards a significant decrease in postprandial triacylglycerol concentration following the moderate fat mixed meal; however when postprandial triacylglycerol concentrations were corrected for their corresponding fasting concentration, no decrease was observed. Despite the improvement in triacylglycerol concentration there was no reduction in fasting or postprandial chylomicron particle number. Insulin sensitivity, measured by HOMA score, and NEFA levels in either the fasting or postprandial states were also not altered in this group of subjects.

The extent of the reduction in fasting triacylglycerol concentration observed in the present study was 16% which is comparable to that reported in other studies [9-12]. In the fasting state the majority of circulating triacylglycerol resides associated with VLDL particles, with chylomicron associated triacylglycerol making only a small contribution [13]. Therefore the observed reduction in fasting triacylglycerol concentration is likely due to a reduction in the circulating concentration of VLDL associated triacylglycerol, a notion strengthened by the observation that fasting chylomicron particle number remained unchanged following exercise.

The majority of studies reporting a significant reduction in postprandial triacylglycerol concentration in response to prior exercise have utilised a relatively high (≥ 1 g fat/kg body weight) oral fat load on the postprandial test day [9-11,14,15]. However the physiological relevance of such a fat load (representing >60% fat by energy intake) to a typical western diet is questionable. With this in mind in the present study we have chosen an oral fat load containing a moderate fat content (0.44 g/kg body weight; 45% total energy) being more typical of a meal that may be consumed as part of a western diet. Our analysis of the postprandial triacylglycerol concentrations following exercise revealed the total area under the postprandial triacylglycerol curve was reduced (with a trend towards significance). However when corrected for fasting triacylglycerol concentration, as measured by incremental area under the postprandial triacylglycerol curve no change was observed following exercise. Hence the reduction in total area under the postprandial triacylglycerol curve results primarily from the decrease in fasting triacylglycerol concentration. A similar observation was made by Kolifa et al [16] who also utilised a fat load of moderate fat content (0.66 g/kg body weight; 35% total energy). In contrast, studies where a higher fat load (≥ 1 g/kg body weight) was given, have reported changes in both the total and incremental areas under the postprandial triacylglycerol curves following exercise [9-11,14,15].

An improved action of lipoprotein lipase (LPL) has been proposed as a mechanism whereby triacylglycerol concentrations are reduced following exercise. Indeed LPL activity is higher in endurance trained athletes compared to sedentary controls [17,18]. However improvements in LPL activity following a single session of moderate intensity exercise are lower in magnitude [19] and may only contribute to part of the observed reductions in postprandial lipaemia [20]. In light of the present findings we can speculate about the involvement of an increased LPL activity on circulating triacylglycerol concentration. If we assume that LPL activity is limiting the hydrolysis of VLDL particles, and that following exercise an increased LPL activity ensues we could envisage that an improved lipolysis of VLDL particles may account for the reductions in fasting triacylglycerol concentration observed. However, if these assumptions are examined in the postprandial state, when the load of triacylglycerol rich lipoproteins is increased, we would also expect to see a reduction in chylomicron associated triacylglycerol concentration, particularly as there is evidence that chylomicrons are hydrolysed preferentially over VLDL [21,22]. However our findings suggest that this is not the case as an improved hydrolysis of chylomicron-associated triacylglycerol would likely result in a reduction in postprandial chylomicron particle number following exercise (assuming chylomicron remnant uptake is not limiting). In other studies the effect of prior exercise on chylomicron metabolism has produced variable results. It has been reported that prior exercise is associated with a reduction in chylomicron concentration in a chylomicron rich (Sf>400) lipoprotein fraction postprandially either by measuring triacylglycerol concentration [15] or particle number (apo B48) [8]. However in another study, prior exercise did not significantly reduce triacylglycerol concentration in this chylomicron rich fraction [11]. A reduction in the concentration of triacylglycerol in VLDL fractions has been consistently reported and represents the majority of the observed reduction in postprandial triacylglycerol concentration [8,11,15]. In the present study we have measured chylomicron particle number directly in serum, hence allowing us to determine the total concentration of chylomicron particles in all lipoprotein fractions. Our observation that prior exercise does not reduce fasting or postprandial concentration of apo B48 is consistent with the above findings and suggests that the majority of the triacylglycerol lowering effect of exercise involves reductions in VLDL-associated triacylglycerol. Chylomicron clearance may be enhanced by exercise but only when the postprandial fat load is greater.

It was first documented that exercise improves insulin sensitivity by Richter et al [23] and given the role that insulin plays in several aspects of lipid metabolism such as increasing LPL activity in adipose tissue [24], inhibiting the hepatic synthesis and release of VLDL [25] and stimulation of LDL-receptor expression [26] it is possible that the effects of exercise on lipid metabolism are mediated at least in part by an increased insulin sensitivity. However in this study we observed that following the exercise session there was no change in fasting insulin or glucose concentration, or postprandial insulin concentration. Results from other studies have been variable; some reporting a reduction in fasting and/or postprandial insulin [11,12,27] and others no change in either fasting or postprandial insulin following exercise [9,16]. It has also been reported that despite the observation of a significant reduction in fasting and postprandial insulin concentrations following exercise there was no correlation between the extent of this decrease and the extent of the decrease in fasting or postprandial triacylglycerol concentrations [27].

## Conclusion

In conclusion, we found that the concentration of pro-atherogenic chylomicra and postprandial chylomicron response was not influenced by a single session of moderate intensity exercise. This finding contrasts with the significantly lower concentration of fasting plasma triglyceride observed following a single session of exercise. We found no evidence that moderate intensity exercise significantly reduced postprandial triacylglycerolemia following a physiologically relevant dose of ingested fat. Collectively, our results and those of others show that triacylglycerol is a poor surrogate marker of chylomicron homeostasis which may be important when considering cardiovascular risk factors.

## Methods

### Subjects

Eight healthy male and female subjects, not overweight or obese, aged 29.8 ± 2.0 years were recruited (n = 5 male, and 3 female). Exclusion criteria included smoking within two years prior, liver or endocrine dysfunction, malabsorption syndrome, anaemia, hypothyroidism, and the use of lipid lowering or hypertensive agents. Diabetes was excluded based on fasting serum glucose being less than 7 mM. Subjects with total serum cholesterol greater than 6 mM or LDL-cholesterol greater than 4 mM were excluded to avoid the potential confounder of genetic hyperlipidemia. We also screened to exclude insulin resistant (assessed by homeostasis model assessment (HOMA) see below; HOMA score >2) [28], overweight or obese (BMI >25 kg/m^2^; waist circumference >100 cm). Informed consent was obtained from all subjects. All procedures were approved by the Human Ethics Committee, Curtin University and conformed to the Helsinki Declaration.

### Design

The study was a randomised crossover design where subjects were required to attend two postprandial testing days. In the afternoon of the day prior to one of the postprandial testing days the subjects performed 90 minutes of moderate intensity exercise. On the day preceding the control postprandial test day the subjects did not perform any exercise. For the female subjects there was an interval of four weeks between each postprandial test day to minimise the confounding effects of menstrual status on lipid metabolism [29].

### Dietary Standardisation

There is evidence that a subject's prior diet may influence the ensuing fasting and postprandial lipid measurements [30,31]; it is therefore important to standardise their dietary intake. In this study we provided the subjects with a standardised meal based on the Australian Guide to Healthy Eating [32], allowing for variations in subject's energy needs by providing a range of "snack" options. This diet was provided for the three days prior to the postprandial test days. All the food to be consumed for each of the three days was pre-packaged and given to the subjects in order to increase dietary compliance. During the first dietary standardisation period the subjects completed a dietary checklist and the meal composition and timing was replicated exactly on the second occasion. Furthermore on the evening prior to the postprandial testing a standard low-fat evening meal was consumed at 7:30 pm (<10 g fat). No other food was consumed following the evening meal.

### Exercise Session

At 3 pm on the day prior to one of the postprandial test days, subjects performed a 90 minute moderate intensity exercise session. The exercise consisted of an initial five minutes of treadmill "familiarisation" followed by three 30 minute treadmill sessions with five minutes of rest between each session. Borg Scale ratings of perceived exertion (RPE) were collected every 5 minutes, and based on these results the treadmill incline and speed were adjusted to keep the subject within the ratings 12–14 (which corresponds to "somewhat hard but still feels okay to continue") [33]. In addition heart rate was measured every five minutes (by short range telemetry) using Polar heart rate monitors however subjects were blinded to their heart rate so as not to influence their RPE. Energy expenditure was calculated by formula according to American College of Sports Medicine [34].

Capillary blood samples were taken at 25 minutes during each of the three exercise sessions for blood lactate determination. Briefly 10 to 25 μL of capillary blood was collected via fingerprick for blood lactate measurements. Measurements were conducted employing a dry-chemistry methodology according to the manufacturer's instructions using an Accusport lactate meter (type 1488767, Boehringer Mannheim) and BM-Lactate Test strips.

### Postprandial lipoprotein assessment

Subjects fasted for 12 hours prior to the start of their postprandial lipoprotein assessment day. In the morning upon arrival at the clinical rooms the subject's body weight, height, waist, and hip circumference were measured following standardised procedures and using a single trained observer.

Fasting blood samples were collected prior to the subjects consuming the moderate fat mixed meal. The moderate fat mixed meal consisted of a breakfast cereal (Uncle Toby's Sports Plus™); cream, skim milk, and skim milk powder. The amount of each component given to the subjects was based on their body weight. The macronutrient composition (expressed per kg body weight) was 0.44 g fat, 0.94 g carbohydrate, 0.27 g protein. After the mixed meal, venous blood samples were collected at the following time points: 1 h, 2 h, 4 h, 6 h, and 8 h. Blood samples were collected in vacuum tubes containing clotting activators for isolation of serum. After clotting blood was centrifuged at approximately 2,000 *g *for 10 min at 4°C. Aliquots of sera were collected and either assayed immediately or stored at -80°C for subsequent analysis. Subjects remained in a semi-recumbent position during the study, were allowed bathroom access and were provided with water at all times.

### Lipid, insulin and glucose assays

Serum triacylglycerol and cholesterol were determined by enzymatic colourimetric kits (Randox Laboratories, Antrim, United Kingdom) as per the manufacturer's instructions. HDL cholesterol was determined following precipitation of apo B containing lipoproteins by phosphotungstic acid and MgCl_2_. LDL cholesterol was determined using a modified version of the Friedewald equation [35]. Insulin were quantified using Immulite 2000 insulin assay (Diagnostic Products Corporation, Los Angeles, USA), which is based on an immunometric "sandwich" assay procedure; and glucose by an enzymatic colourimetric kit based on the glucose oxidase method(Randox Laboratories, Antrim, United Kingdom). Non-esterified fatty acids (NEFA) were determined using the Wako NEFA C kit (Wako Pure Chemical Industries, Ltd, Osaka, Japan) according to the manufacturer's instructions.

### Apolipoprotein B48 determination

Serum apo B48 was quantitated using a Western Blotting/Enhanced Chemiluminescence procedure as previously described [36]. Briefly serum samples were prepared and loaded onto Invitrogen Tris-Acetate (3–8%) precast gels and submitted to electrophoresis according to the manufacturer's instructions (Invitrogen, Mount Waverly, Australia). Following separation by electrophoresis, serum proteins were electrophoretically transferred to PVDF membranes (Millipore, North Ryde, Australia). After blocking unbound sites the membranes were incubated with an anti-human apo B antibody (raised in rabbit (DAKO A/S Denmark)), visualisation of the antigen-antibody complex was then achieved following incubation with an anti-rabbit IgG (HRP conjugated) antibody (Amersham, Little Chalfont, UK) and enhanced chemiluminescence reagent (Amersham, Little Chalfont, UK), according to the manufacturer's guidelines. Membranes were exposed to blue-light film (Amersham, Little Chalfont, UK) and developed in an AGFA CP1000 X-Ray Developer (AGFA, Nunawading, Australia). Apo B48 bands were identified and quantitated by densitometry against purified apo B48 protein of known mass using NIH Image (version 1.6.3). The mean intra- and inter-assay coefficients of variance for apo B48 were each less than 4%.

### Quantification of Postprandial Metabolism

Postprandial metabolism was quantified by calculating the area under the curve (AUC) and the incremental area under the curve (IAUC), the latter of which was estimated as the difference between the area defined below the baseline concentration and the area under the serum curve between 0 h and 8 h. The IAUC represents the increase in area after the response of the fat load above fasting concentrations whereas AUC reflects total serum concentrations of these measures over time.

### Measurement of insulin resistance state

Estimation of the subjects' state of insulin resistance was by calculation of a HOMA score as described by Matthews et al [28].

### Statistical analysis

Statistical analysis was by parametric methods using SPSS 11 for Macintosh OS X (SPSS Inc., Chicago, IL). Comparison of fasting and postprandial measures on the days following control and exercise sessions were done using paired *t*-tests. Significance was accepted at the *P *< 0.05 level and data are presented as mean ± SEM.

## Competing interests

The author(s) declare that they have no competing interests.

## Authors' contributions

AJ conceived of the idea for the study, designed and carried out the study, analysed the results, and drafted the manuscript. KSC assisted in carrying out the study, analysis of the data, and drafting of the manuscript. JM assisted in the design of the study, analysis of the results and drafting of the manuscript.
